# Mixed *Cryptosporidium* Infections and HIV

**DOI:** 10.3201/eid1206.060015

**Published:** 2006-06

**Authors:** Vitaliano Cama, Robert H. Gilman, Aldo Vivar, Eduardo Ticona, Ynes Ortega, Caryn Bern, Lihua Xiao

**Affiliations:** * Centers for Disease Control and Prevention, Atlanta, Georgia, USA;; † Johns Hopkins University, Baltimore, Maryland, USA;; ‡ Asociación Benéfica Proyectos en Informática, Salud, Medicina y Agricultura, Lima, Peru;; § Hospital Arzobispo Loayza, Lima, Peru;; ¶ Hospital Dos de Mayo, Lima, Peru;; # University of Georgia, Griffin, Georgia, USA

**Keywords:** Cryptosporidiosis, Cryptosporidium genotypes, AIDS-related opportunistic infections, intestinal diseases, parasitic, HIV wasting syndrome, dispatch

## Abstract

Mixed *Cryptosporidium* infections were detected in 7 of 21 patients with a diagnosis of rare *Cryptosporidium canis* or *C. felis* infections; 6 patients were infected with 2 *Cryptosporidium* spp. and 1 patient with 3 species. Mixed infections may occur more frequently than previously believed and should be considered when assessing cryptosporidiosis.

*Cryptosporidium* spp. infect humans and other vertebrate animals. Persons with compromised immune systems can suffer life-threatening chronic diarrhea, especially when their CD4+ lymphocyte counts fall <200 cells/μL. At least 7 *Cryptosporidium* spp. have been detected in immunocompromised patients (*1*). Nonetheless, the role of concurrent or mixed infections in the pathogenesis and transmission of *Cryptosporidium* spp. is unclear. Mixed infections of *Cryptosporidium hominis* and *C. parvum* have been reported in several patients from Switzerland and England (*2**,**3*). Additional studies from the United Kingdom reported simultaneous infections with these 2 species: 4 cases in 2 waterborne outbreaks and 2 cases of sporadic infections from 1995 to 1999 (*4*). In a more recent study, 12% of 135 clinical specimens from Aberdeenshire, Scotland, had concurrent *C. parvum* and *C. hominis* infections (*5*). Mixed *C. hominis*–*C. parvum* infections were also seen in 2 of 38 archived human specimens in a study conducted in the United States (*6*). These observations suggest that mixed *Cryptosporidium* infections are not uncommon.

Mixed infections may not be readily identified by commonly used molecular diagnostic tools because of preferential polymerase chain reaction (PCR) amplification of the predominant genotypes or the specificity of molecular tools (*6*). For example, PCR–restriction fragment length polymorphism (RFLP) tools based on the small subunit (SSU) rRNA gene are frequently used in genotyping *Cryptosporidium* spp. because they have higher sensitivity and detect more species than PCR-RFLP tools based on other genes (*7*).

Two previous studies in Peru used an SSU-rRNA–based PCR-RFLP tool to genotype *Cryptosporidium* specimens from children (*8*) and AIDS patients (*1*). A variety of *Cryptosporidium* spp. were found in both patient populations; *C. hominis* was the predominant species, followed by *C. parvum*, *C. meleagridis*, *C. canis*, and *C. felis*, but mixed infections were rarely detected (*1**,**8*). However, a recent study of some of the specimens that used PCR tools that selectively amplify DNA of *C. parvum* and closely related species identified concurrent infections of *C. hominis* in specimens previously diagnosed as having only *C. canis*, *C. muris*, or *C. suis* (*7*). Another recent study has shown that an SSU rRNA–based PCR-RFLP tool had only a 31%–74% success rate in detecting concurrent infections with *C. parvum* and *C. hominis* (*9*).

## The Study

We addressed the question of whether Peruvian HIV-positive patients infected with the usual *C. canis* or *C. felis* parasites were co-infected with *C. hominis*, *C. parvum*, or *C. meleagridis* (*7*). The study protocol was approved by the participating institutional review boards. All participants gave written informed consent.

Mixed infections were identified by using 2 PCR-RFLP tools that only amplify *C. hominis*, *C. parvum*, or *C. meleagridis* (*7*). One tool was based on the dihydrofolate reductase (DHFR) gene (*10*) and the other on the *Cryptosporidium* oocyst wall protein (COWP) gene (*11*). Fifty-six stool specimens from 21 HIV-infected persons with previous diagnoses of *C. canis* or *C. felis* with an SSU rRNA–based PCR-RFLP tool were re-analyzed with these 2 molecular tools. DNA was extracted by using the QIAamp stool DNA extraction kit (Qiagen Inc., Valencia, CA, USA), and 1 μL DNA was used in nested PCR analyses of the DHFR and COWP genes. Secondary PCR products positive for *Cryptosporidium* were digested with restriction enzymes *BpuA* I for the DHFR tool or *Rsa* I for the COWP tool (*10**,**11*). Results of RFLP diagnosis were confirmed by DNA sequence analysis. All secondary PCR products were sequenced with a 3100 ABIPrism Genetic Analyzer (Applied Biosystems, Foster City, CA, USA). The sequences obtained were aligned with reference sequences from GenBank by using BioEdit version 7.0.5 (Isis Pharmaceuticals, Carlsbad, CA, USA).

The PCR analysis of both DHFR and COWP genes showed that 17 specimens from 7 patients yielded products of the expected size for *Cryptosporidium* spp. (Figure, panel A, and Table). Restriction analysis of DHFR products with *BpuA* I showed that 4 patients had banding patterns indicative of *C. hominis*, 1 patient had the pattern of *C. parvum*, 1 patient had the pattern of *C. meleagridis*, and 1 patient had the patterns of *C. hominis* and *C. meleagridis* (Figure, panel B). Likewise, RFLP analysis of the COWP PCR products digested with *Rsa* I showed 3 banding patterns that were in complete agreement with the results obtained for the DHFR PCR-RFLP tool (Figure, panel C). Therefore, 2 of the 12 *C. canis*–infected patients had *C. hominis*, 1 had *C. parvum* and 1 had both *C. hominis* and *C. meleagridis*; of the 9 *C. felis*-infected patients, 2 had *C. hominis* and 1 had *C. meleagridis* (Table).

**Figure:**
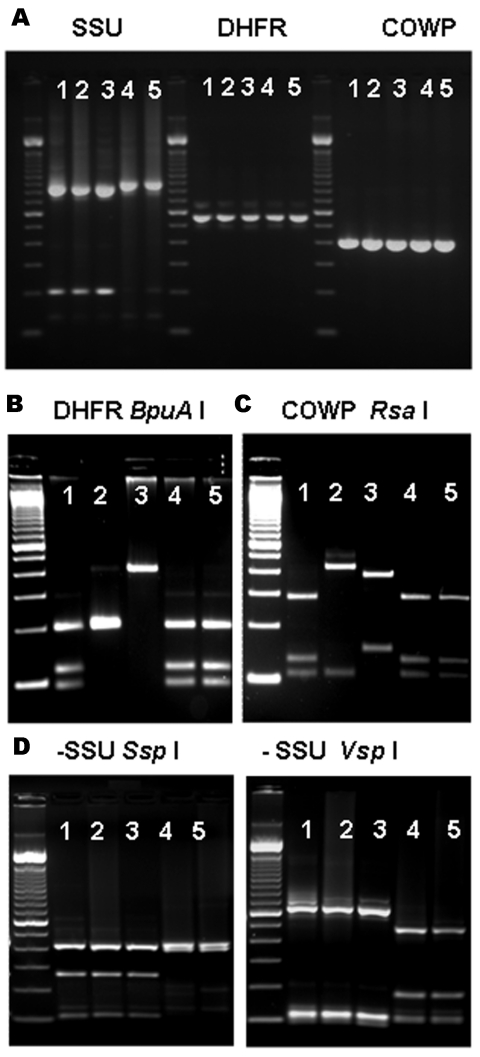
Multilocus polymerase chain reaction–restriction fragment length polymorphism (PCR-RFLP) analysis of specimens previously identified as *Cryptosporidium canis* and *C. felis*. A) Agarose gel electrophoresis of PCR-amplified products of specimens previously identified as *C. canis* (lanes 1–3) and *C. felis* (lanes 4 and 5) with molecular tools based on the small subunit (SSU) rRNA, dihydrofolate reductase (DHFR), and *Cryptosporidium* oocyst wall protein (COWP). Molecular markers in all photos are 100-bp ladders. B) RFLP analysis of DHFR-based PCR amplification products using *BpuA* I restriction enzyme; lanes 1, 4, and 5 are *C. hominis*; lane 2 is *C. parvum*; and lane 3 is *C. meleagridis*. C) RFLP analysis of COWP-based PCR amplification products using *Rsa* I restriction enzyme; lanes 1, 4, and 5 are *C. hominis*; lane 2 is *C. parvum*; and lane 3 is *C. meleagridis*. D) RFLP analysis of the SSU-based PCR products using restriction enzymes *Ssp* I (left) and *Vsp* I (right); the combined patterns for lanes 1 to 3 correspond to *C. canis* and lanes 4 and 5 to *C. felis*.

**Table:** Results of multilocus genotyping of *Cryptosporidium* specimens originally diagnosed as *Cryptosporidium canis* and *C. felis* by an SSU rRNA–based PCR-RFLP tool*

Participant	No. specimens tested	No. days between first and last specimen	*Cryptosporidium* genotype by locus (no. specimens)			
			SSU rRNA	COWP	DHFR	Mixed infection
0043D	7	29	*C. canis*	–	–	No
0214D	2	5	*C. canis*	–	–	No
0448D	4	45	*C. canis*	*C. hominis* (1) and *C. meleagridis* (2)	*C. hominis* (2) and *C. meleagridis* (2)	Yes
1083D	1	–	*C. canis*	–	–	No
1322D	2	2	*C. canis*	–	–	No
0002D	1	–	*C. canis*	–	–	No
0034D	7	56	*C. canis*	*C. parvum* (2)	*C. parvum* (2)	Yes
0482D	1	–	*C. canis*	*C. hominis* (1)	*C. hominis* (1)	Yes
0500D	1	–	*C. canis*	–	–	No
0533D	3	3	*C. canis*	–	–	No
0670D	4	414†	*C. canis*	*C. hominis* (4)	*C. hominis* (2)	Yes
0725D	1	–	*C. canis*	–	–	No
0044A	1	–	*C. felis*	*C. meleagridis* (1)	*C. meleagridis* (1)	Yes
0076A	4	31	*C. felis*	*C. hominis* (3)	*C. hominis* (1)	Yes
0668A	3	3	*C. felis*	*C. hominis* (1)	*C. hominis* (2)	Yes
0673A	5	31	*C. felis*	–	–	No
0817A	2	2	*C. felis*	–	–	No
0891A	1	–	*C. felis*	–	–	No
1344A	3	3	*C. felis*	–	–	No
0569D	2	2	*C. felis*	–	–	No
0776D	1	–	*C. felis*	–	–	No

*PCR-RFLP, polymerase chain reaction–restriction fragment length polymorphism; SSU, small subunit; COWP, *Cryptosporidium* oocyst wall protein; DHFR, dihydrofolate reductase gene. †Specimens correspond to 2 visits 14 months apart.

All DHFR and COWP PCR products were sequenced, which confirmed the results of the RFLP diagnosis. Altogether, 8, 2, and 3 DHFR sequences were obtained for *C. hominis*, *C. parvum*, and *C. meleagridis*, respectively. The *C. hominis* and *C. meleagridis* DHFR sequences were identical to XM_660774 and AY391725, respectively. The *C. parvum* DHFR sequences were homologous to XM_625460, with an insertion at position 37 and 4 bp substitutions at positions 66, 69, 364, and 367. Likewise, 10, 2, and 3 COWP sequences were obtained for *C. hominis*, *C. parvum*, and *C. meleagridis*, respectively, and were identical to AF481960, AF266273, and AY166840, respectively, in GenBank. The *C. parvum* DHFR nucleotide sequence obtained from this study is deposited in GenBank under accession no. DQ352814.

To confirm the original diagnosis of *C. canis* and *C. felis* infection, we reanalyzed all DNA preparations of these specimens with the SSU rRNA genotyping tool (*7*). Results were in complete agreement with those obtained previously (*7*): 19 specimens from 12 patients had *C. canis*, 15 specimens from 9 patients had *C. felis*, and no specimens had mixed *Cryptosporidium* spp., as indicated by RFLP patterns (Table and Figure panel D).

Data on diarrhea at study enrollment were available for 4 of the 7 patients with mixed infections and all 14 patients without mixed infections. Among persons with mixed infections, 1 did not have diarrhea, 2 had diarrhea lasting <30 days, and 1 had diarrhea >5 months. Seven of 14 patients without mixed infections had diarrhea: 5 had acute diarrhea lasting <30 days, and 2 had chronic diarrhea lasting >5 months (difference in prevalence of diarrhea for mixed versus single infections was not significant by the Fisher exact test). The average CD4+ lymphocyte count among the patients with mixed infections was 130 cells/μL. Of the 7 patients with mixed infections, 3 had specimens collected >30 days after the first detection, and mixed infections with the same species were still identified. The persistence of 2 species for >1 month is in contrast to a report that 1 *Cryptosporidium* genotype rapidly displaces the other during experimental infections of animals (*6*).

## Conclusions

Concurrent infection with multiple *Cryptosporidium* spp. may affect clinical manifestations since *C. hominis* and *C. parvum* induce different sequelae in humans (*12*). The frequent finding of *C. hominis* in *C. canis*– and *C. felis*–infected persons also raises the question of infection sources. Although these 2 species are traditionally associated with animals, anthroponotic transmission may play a role in their acquisition in humans. Recent analyses demonstrate that a large proportion of human infections with *C. parvum*, another traditional zoonotic species, are actually due to anthroponotic transmission (*13**,**14*).

Our results also suggest that although the SSU rRNA–based PCR-RFLP tool or similar PCR techniques can detect and differentiate a wide range of *Cryptosporidium* species or genotypes, their usefulness in detecting mixed infections was compromised by preferential PCR amplification of the dominant species or genotype in specimens. This problem is likely inherited with most PCR tools. Thus, the use of PCR tools with broad specificity in combination with species-specific tools is needed to address the issue of mixed *Cryptosporidium* infections.

Our findings demonstrate that mixed infections are more frequent and persist longer in HIV-infected patients than previously believed. The clinical importance of these findings is not clear because of the study's cross-sectional nature. Future studies should employ tools that can detect mixed *Cryptosporidium* infections in longitudinal studies, evaluate the frequency of mixed infections of *C. hominis* and *C. parvum*, and assess their clinical and epidemiologic implications in both immunocompetent and immunocompromised persons.
